# Effects of the Edaravone, a Drug Approved for the Treatment of Amyotrophic Lateral Sclerosis, on Mitochondrial Function and Neuroprotection

**DOI:** 10.3390/antiox11020195

**Published:** 2022-01-20

**Authors:** Sun Joo Cha, Kiyoung Kim

**Affiliations:** 1 Department of Medical Sciences, Soonchunhyang University, Asan 31538, Korea; cktjswn92@sch.ac.kr; 2 Department of Medical Biotechnology, Soonchunhyang University, Asan 31538, Korea

**Keywords:** edaravone, oxidative stress, antioxidant, mitochondria, neurodegenerative disease, amyotrophic lateral sclerosis

## Abstract

Edaravone, the first known free radical scavenger, has demonstrated cellular protective properties in animals and humans. Owing to its antioxidant activity, edaravone modulates oxidative damage in various diseases, especially neurodegenerative diseases. In 2015, edaravone was approved in Japan to treat amyotrophic lateral sclerosis. The distinguishing pathogenic features of neurodegenerative diseases include high reactive oxygen species levels and mitochondrial dysfunction. However, the correlation between mitochondria and edaravone has not been elucidated. This review highlights recent studies on novel therapeutic perspectives of edaravone in terms of its effect on oxidative stress and mitochondrial function.

## 1. Introduction

The incidence and prevalence of amyotrophic lateral sclerosis (ALS) are increasing worldwide. The prevalence of ALS varies among countries. According to recent studies in Europe, the global ALS incidence and prevalence were reported as 0.6–3.8 and 4.1–8.4 per 100,000 persons, respectively. In Europe, ALS prevalence was estimated to be higher than 10–12 per 100,000 persons, with an incidence of 1.75–3 per 100,000 persons per year [1]. In the United States, as per the National ALS Registry, the estimated prevalence of ALS cases in 2015 was 5.2 per 100,000 persons [2]. The prevalence of ALS in South Korea in 2015 was 3.43 per 100,000 persons [3]. On account of these statistics, there is an urgent need to develop appropriate methods for the treatment and management of ALS. ALS is characterized by progressive degeneration of both upper and lower motor neurons. To date, two ALS treatment agents have been approved: riluzole and edaravone. Riluzole was the first treatment agent for ALS approved in 1995 by the FDA. It exerts an anti-glutamatergic effect by blocking the release of glutamate at the presynaptic terminus and inactivating voltage-dependent sodium channels [4]. However, riluzole is associated with side effects, such as liver disease, diarrhea, and mild nausea [5]. In this review, we discuss edaravone as an alternative treatment agent for ALS.

Edaravone (MCI-186, 3-methyl-1 phenyl-2-pyrazolin-5-one) is the first described free radical scavenger. It is a colorless transparent liquid, and its acid dissociation constant pKa is 7.0, indicative of weak acidity. It has strong free radical scavenging activity and can interact with both hydroxyl and peroxyl radicals to undergo oxidation. It was originally developed by Mitsubishi Chemical Industries Ltd. to treat neuropathy caused by cerebral infarction [6,7]. Edaravone has been used in Japan since 2001 for the treatment of acute ischemic stroke [8]. In 2011, Mitsubishi started phase III clinical trials for edaravone as an ALS treatment agent, leading to its approval in Japan in 2015. Edaravone was also used as a therapeutic drug for ALS in South Korea since 2015. It was approved in the US by the FDA in 2017 and in Canada in 2018 [9].

## 2. Role of Edaravone under Oxidative Stress

Edaravone is a free radical scavenger, which offers neuroprotection under oxidative stress. Oxidative stress plays a critical role in the pathogenesis of several neurodegenerative diseases, including ALS, Parkinson’s disease (PD), Alzheimer’s disease (AD), and Huntington’s disease. Oxidative stress creates an imbalance in the cellular system, leading to the production of free radicals and peroxides [reactive oxygen species (ROS), and reactive nitrogen species (RNS)], as well as disrupted proteins, lipids, and DNA [10]. Among these, ROS produced in the mitochondria plays a significant role in molecular and cellular systems. Under redox conditions, cells produce excessive ROS or free radicals, which are scavenged or neutralized by antioxidant systems. Excessive ROS production or defective antioxidant defense systems are associated with various diseases, especially neurodegenerative diseases [11,12]. Thus, antioxidants play an important role in combating oxidative stress to prevent disease development.

### 2.1. Neuroprotective Effect of Edaravone

Several studies have reported the neuroprotective properties of edaravone. For instance, treatment with edaravone in a rat model of traumatic brain injury (TBI) prevented hippocampal CA3 neuron loss, decreased oxidative stress, and reduced programmed death of neuronal cells. Moreover, edaravone also exerted protective effects on non-neuronal cells, such as decreased astrocyte and glial activation [13]. In a rat model of cerebral infarction, edaravone reduced the amount of malondialdehyde-thiobarbituric acid adduct in a dose-dependent manner [14]. Furthermore, edaravone treatment inhibited rotenone-mediated degeneration of dopaminergic neurons in the midbrain of a rotenone-induced rat model of PD and also reduced ROS [15]. Injection of edaravone in ALS-mimicking mutant SOD1 G93A mice notably slowed the degeneration of motor neurons. Additionally, in mice administered with a high dose of edaravone, deposition of mutant SOD1 in the spinal cord was greatly reduced [16]. Furthermore, edaravone decreased ROS production, apoptosis, and aggregation in an in vitro Aβ_25–35_-induced AD model [17]. Based on these collective findings, many researchers have suggested that edaravone offers neuroprotection against oxidative stress in various neurological diseases, including ALS. Here, we reviewed edaravone as a potential treatment for ALS.

### 2.2. Mechanisms of Edaravone Action

Understanding antioxidant defense signals and enzymes in human diseases is important for drug development. Nuclear factor erythroid 2-related factor-2 (Nrf2) is well known as a stimulator of antioxidant activities against oxidative stress, which damages antioxidant and detoxification enzymes. Upon activation by inflammation or injury, Nrf2 is translocated from the cytoplasm to the nucleus, where it binds to antioxidant response elements in the promoter region of various antioxidant and detoxifying enzymes, such as heme oxygenase-1 (HO-1) and NAD(P)H quinone oxidoreductase-1 (NQO1), and thus defends cells against oxidative stress injury [18].

Treatment with edaravone in rats with cerebral infarction resulted in notably high expression of Nrf2 and HO-1, the latter being a crucial endogenous antioxidant, and activated essential anti-oxidant defense system in the brain. Furthermore, edaravone treatment resulted in weight gain, enhanced neurological function, and reduced brain tissue water content and blood-brain-barrier permeability [19]. Edaravone has also been reported to exert a protective effect against lung injury by eliminating ROS. Edaravone treatment in an experimental mouse model of asthma significantly decreased the levels of oxidative stress markers and recovered SOD1 levels. Moreover, edaravone treatment increased Nrf2 expression and decreased kelch-like ECH-associated protein-1 (Keap1) expression, resulting in a marked reduction in the Keap1/Nrf2 ratio. HO-1 levels were also increased following edaravone treatment. Thus, edaravone exerts an anti-asthmatic effect by activating the Keap1/Nrf2 pathway and increasing HO-1 expression [20].

Another study suggested that edaravone exerts neuroprotective effects by increasing neuron density and reducing neuronal damage induced by kainate. In addition, edaravone treatment suppressed the downregulation of Nrf2 and HO-1-induced by kainate while decreasing NF-κB, proinflammatory cytokines, and inflammatory protein levels in the hippocampus [21]. Similarly, edaravone reversed chlorpyrifos-induced neuronal toxicity by activating the Nrf2 signaling pathway [22]. Edaravone treatment was found to reverse the downregulation of Nrf2 and nuclear translocation induced by Aβ_25–35_. Moreover, knockdown of Nrf2 led to a decrease in SOD and HO-1 expression, and reduced the neuroprotective effect of edaravone against Aβ_25–35_ [23]. Therefore, Nrf2 is one of the therapeutic targets of edaravone under oxidative stress in neurodegenerative diseases.

### 2.3. Effect of Edaravone in Patients with ALS

As mentioned previously, edaravone was approved as a treatment for ALS in Japan in 2015. Edaravone has also been approved as an ALS treatment in other countries, including South Korea, the United States, and Canada [9]. In phase II clinical trials, ALS patients treated with 60 mg edaravone showed a reduction in the levels of free acid and markers for oxidative stress compared to those treated with the placebo, suggesting antioxidant activity of edaravone [24]. Edaravone was found to be efficient in a small subset of ALS patients who met the criteria identified in the post-hoc analysis of a previous phase III study, showing a notably smaller decrease in the Revised ALS Functional Rating Scale (ALSFRS-R) score compared with the placebo [25]. Moreover, edaravone is beneficial in ALS patients even after receiving a placebo, and its efficacy is preserved for up to 1 year as assessed by the ALSFRS-R score [26]. Additionally, edaravone was shown to be an effective drug for ALS patients in the early stages of clinical trials by analyzing the forced vital capacity and the rate of decline of the ALSFRS-R score. Post-marketing experience of edaravone in South Korea and Kuwait was appropriately favorable, with effective results in reducing the progression of ALS disease. Nevertheless, edaravone showed no significant effects in ALS patients in Italy and Israel. In the United States and Argentina, information on the efficiency of edaravone is still limited [27]. Therefore, more post-marketing experience of edaravone and its effectiveness in clinical trials are needed.

## 3. Importance of Mitochondrial Function in ALS

ALS, one of the subtypes of motor neuron disease, is characterized by progressive deterioration of both upper and lower motor neurons. ALS causes muscle atrophy and paralysis and eventually death due to respiratory failure. The global prevalence of ALS is 1–6 per 100,000 people and varies across countries [28]. Most patients with ALS exhibit no clear evidence of genetic susceptibility and appear to have sporadic ALS (90–95%) caused by environmental risk factors. The remainder comprises patients with familial ALS caused by mutations in specific genes. Over 20 genes related to ALS pathogenesis have been identified, including *SOD1*, transactive response DNA-binding protein (*TDP-43*), fused in sarcoma/translocated in liposarcoma protein (*FUS/TLS*), and *C9orf72* [29]. Various molecular pathogenic mechanisms are involved in motor neuron degeneration, including RNA metabolic defects, proteostasis imbalance, damaged axonal transport, and oxidative stress [30].

In particular, one of the pathogenic mechanisms associated with ALS is mitochondrial damage [31]. Mitochondria play a critical role in cell metabolism as generators of ATP via oxidative phosphorylation. However, dysfunctional mitochondria in ALS show abnormal morphology, resulting in swollen, fragmented, aggregated, or vacuolated mitochondria. For example, overexpression of wild-type TDP-43 or ALS-linked TDP-43 mutants A315T, Q331K, and M337V is associated with fragmented, vacuolated, and aggregated mitochondria [32,33,34]. Moreover, abnormal morphological clusters of mitochondria were found along the axon in SOD1 G93A mice [32]. ALS-associated FUS mutants (R521G and R521H) were associated with significantly shortened mitochondria in motor neurons [35]. Additionally, overexpression of the wild-type FUS resulted in an imbalance in mitochondrial dynamics, resulting in fragmented mitochondria [36,37].

Further, mitochondrial damage is induced by interaction with ALS-related proteins. SOD1 mutants directly interact with voltage-dependent anion channel 1 (VDAC1), which plays a role in exchanging ATP and ADP, thereby inhibiting channel transfer and ADP permeability [38]. The accumulation of ALS-linked TDP-43 mutations was also found in mitochondria; these bind to mRNAs encoding ND3 and ND6 that form composites of complex I, damage their transcription, and cause complex I disassembly [39]. Furthermore, wild-type FUS and FUS P525L mutants interact with the mitochondrial chaperone, the heat shock protein of 60 kDa (HSP60), mediating FUS translocation from the cytoplasm to the mitochondria [40]. Reduction in mitochondrial respiration and ATP production is the predominant feature of mitochondrial dysfunction in ALS. SOD1 G93A transgenic mice showed decreased complex I activity [41]. Depolarization of mitochondria accompanied by reduction of complex I activity was observed in NSC-34 cells expressing wild-type or mutant TDP-43 (Q331K and M337V) [34,42,43]. Expression of wild-type or ALS mutant FUS (R521C and R518K) in NSC-34 cells hindered ATP production [44]. Moreover, lymphocytes in patients with sporadic ALS showed reduced complex I activity and ATP levels [45].

Other factors in mitochondrial dysfunction include an imbalance of calcium homeostasis and reduction of contact between ER and mitochondria. Reduced ER interaction with mitochondria in ALS-related TDP-43 and FUS was reported to be caused by the downregulation of GSK3β-dependent VAPB-PTP1P51 interaction. Mutations of TDP-43 and FUS result in decreased calcium absorption in the mitochondria and an associated increase in cytosolic calcium, triggering calcium release from the ER [44,46]. SOD1 G93A transgenic mice also exhibit markedly reduced mitochondrial calcium levels [47].

Balanced mitochondrial dynamics are important during mitochondrial fusion and fission. Mitochondrial fusion plays a role in the exchange of metabolites, DNA, and proteins in mitochondria, while mitochondrial fission plays a role in the isolation and removal of the impaired mitochondrial parts through mitophagy. Dynamin-related protein 1 (Drp1) and fission 1 (Fis1) regulate mitochondrial fission, while mitofusin 1 (Mfn1), mitofusin 2 (Mfn2), and optic atrophy 1 (Opa1) regulate mitochondrial fusion. Several reports suggest that imbalanced mitochondrial dynamics contribute to ALS pathogenesis. Expression of SOD1 G93A in SH-SY5Y and NSC-34 cells decreased Opa1 levels and increased Drp1 levels, resulting in fragmented mitochondria; whereas, SOD1 G93A transgenic mice showed decreased Mfn1 and Opa1 and increased Drp1 levels [48,49]. TDP-43, FUS, and TAF15-mediated ALS decreased the level of Marf, a homolog of mitofusins in *Drosophila*, leading to an imbalance in mitochondrial dynamics [36]. The level of Fis1 was markedly increased in TDP-43 A382T patient-derived fibroblasts [50].

Impaired axonal transport of mitochondria is a well-known phenotype in ALS. Axonal transport of mitochondria is regulated by the outer mitochondrial membrane protein Miro1 [51]. Miro1 levels were decreased in SOD1 G93A and TDP-43 M337V transgenic mice, as well as in the spinal cord of patients with ALS [52]. Thus, damaged axonal transport of mitochondria may result in defective ATP generation and calcium absorption at synaptic terminals.

Mitochondrial dysfunction, including damaged morphology, imbalanced mitochondrial dynamics, defective calcium homeostasis, impaired mitochondrial respiration, and reduction of ATP, are features of ALS pathogenesis. Therefore, potential drugs and other therapeutic approaches for ALS need to restore mitochondrial function.

## 4. Role of Edaravone in Mitochondria

Mitochondria play an important role in cell metabolism and survival. Dysfunction of mitochondria is the primary pathogenic feature in various neurological diseases, such as AD, PD, ALS, and TBI. Many studies have demonstrated the effects of edaravone as an antioxidant. This review focuses on the effects of edaravone in the mitochondria of cells (Table 1) and animal models (Table 2).

### 4.1. In Vitro Models

AD is a progressive neurodegenerative disease characterized by extracellular amyloid senile plaques and intracellular aggregates of the hyperphosphorylated tau protein. Aβ-induced oxidative stress generates mitochondrial ROS. The loss of mitochondrial membrane potential, high levels of apoptosis and ROS, decreased ATP levels, and swollen mitochondrial morphology appears in Aβ_25–35_-induced SH-SY5Y cells. Exposure to edaravone alleviated these mitochondrial defects induced by Aβ_25–35_ [53]. In Aβ_25–35_-induced PC12 cells, mitochondrial peroxidation, ROS levels, and apoptosis were highly increased. However, intracellular glutathione and SOD concentrations were markedly decreased when PC12 cells were co-treated with edaravone and Aβ_25–35_. Additionally, oxidative damage was reduced [17]. N2a/Swe.Δ9 cells, an AD cell model, exhibited decreased mitochondrial membrane potential and cell viability. However, edaravone treatment inhibited this observed reduction in mitochondrial membrane potential and thereby increased the ROS levels. The mitochondria-dependent apoptosis pathway activated through decreased Bax/Bcl-2 ratio was inhibited in edaravone-treated N2a/Swe. Δ9 cells. Edaravone also ameliorated the release of cytochrome c and suppressed caspase-3 activation in N2a/Swe. Δ9 cells [54]. Therefore, edaravone exerted neuroprotective effects in an AD-related model and may offer a potential complementary function for AD treatment.

PD, the second most common neurodegenerative disorder after AD, is characterized by progressive degeneration of dopaminergic neurons. Several genes, including Lrrk2, Park2, PINK1, and DJ-1, are associated with the pathogenesis of PD [55]. Wild-type DJ-1 protects against cytotoxicity induced by oxidative stress [56]. The L166P mutant of DJ-1 is a loss-of-function mutation associated with early-onset familial PD. L166P mutation of DJ-1 results in raised oxidative stress and mitochondrial dysfunction in N2a cells. However, treatment with edaravone modulates the mitochondria-dependent apoptosis pathway in mutant DJ-1 L166P N2a cells. Furthermore, edaravone increases the expression of vesicular monoamine transporter 2 (VMAT2), the central neuronal system vesicular transporter, in N2a cells in a dose-dependent manner [57]. VMAT2 is an H^+^-ATPase antiporter that transports dopamine, serotonin, norepinephrine, epinephrine, and histamine into small and dense core synaptic vesicles for their release from neurons. Originally, VMAT was identified due to its capability to defend cells from MPP^+^ toxicity, a metabolite of dopamine toxic substances, and MPTP-induced PD model [58,59]. Moreover, MPP^+^-treated PC12 cells showed mitochondrial fragmentation, leading to an increase in Drp1 and Fis1 protein levels and a decrease in Mfn2 and Opa1 protein levels. Edaravone recovered the protein levels of Drp1, Mfn2, and Opa1 in MPP^+^-treated PC12 cells. These results suggest that edaravone exerts a protective effect against mitochondrial fusion disturbance induced by MPP^+^ in PC12 cells via downregulation of Drp1 and upregulation of Opa1/Mfn2 [60]. Rotenone, which is a specific inhibitor of mitochondrial complex I, induces ROS production and is used to develop PD models [61]. In rotenone-induced SH-SY5Y cells, the expression of genes related to mitochondrial biogenesis and mitochondrial dynamics was found to be decreased. However, edaravone suppressed this reduction [62].

Evidence suggests that edaravone plays a neuroprotective role against oxidative stress by modulating mitochondrial function. For instance, edaravone treatment increased mitochondrial membrane potential and suppressed nuclear translocation of Nrf2 in sodium nitroprusside-induced PC12 cells [63]. Paraquat is a highly toxic, common herbicide. Treatment with edaravone increased mitochondrial viability and decreased ROS production in paraquat-treated A549 cells [64]. Oxygen glucose deprivation in PC12 cells resulted in swollen mitochondria and decreased mitochondrial membrane potential. Edaravone treatment inhibited this loss of mitochondrial membrane potential and reversed defective mitochondrial morphology [65]. Therefore, using in vitro disease and non-disease models, the anti-oxidant effect of edaravone as well as its role in modulating mitochondrial function was confirmed.

**Table 1:** Studies that used edaravone in in vitro models of mitochondrial diseases.

Derived Tissue	Associated Disease	Experimental Model	Action of Edaravone in Mitochondria									Ref.
			ΔY	ATP Level	Peroxidation	ROS	Morphology	Fission/Fusion	Biogenesis	Mptp Opening	Apoptosis	
Bone, Marrow	AD	Aβ_25-35_-induced SH-SY5Y cell	Increase	Increase	-	-	Preserve	-	-	-	-	[53]
	PD	Rotenone-treated SH-SY5Y cell	-	-	-	-	-	Up-regulation of Drp1 and Mfn2	Up-regulation of PGC-1α and TFAM	-	-	[62]
Adrenal gland	AD	Aβ_25-35_-treated PC12 cell	-	-	Decrease	-	-	-	-	-	-	[17]
	PD	MPP^+^-treated PC12 cell	-	-	-	-	-	Up-regulation of Opa1/Mfn2, Down-regulation of Drp1	-	-	-	[60]
		Sodium nitroprusside-induced PC12 cell	Increase	-	-	-	-	-	-	-	-	[63]
		Oxygen glucose deprivation in PC12 cell	Increase	-	-	Decrease	Preserve	-	-	-	-	[65]
Brain	AD	N2a/Swe.Δ9 cell	Increase	-	-	-	-	-	-	-	Inhibit	[54]
	PD	L166P mutant of DJ-1 in N2a cell	Increase	Increase	-	-	-	-	-	-	Inhibit	[57]
Umbilical cord		Iron overload injury HUVECs	Increase	-	-	-	-	-	-	Inhibit	-	[66]
		Doxorubicin-induced HUVECs	Increase	-	-	Decrease	-	-	-	Inhibit	Inhibit	[67]
Lung		Paraquat-treated A549 cell	-	-	-	Decrease	-	-	-	-	-	[64]

### 4.2. Ex Vivo Models

Recent studies using ex vivo experiments have reported that edaravone is related to neurodegenerative disorders. The activation of microglia is a critically important pathological feature in AD patients. Aβ-induced pro-inflammatory response, such as NLRP 3 inflammasome leading to caspase-1 activation and IL-1β release, was inhibited by edaravone treatment. Edaravone also attenuated the depolarization of mitochondrial membrane and reduced mitochondria-derived ROS production in Aβ-treated microglia [68].

Furthermore, edaravone reversed swollen mitochondria isolated from the rat brain that resulted from the overloading of Ca^2+^ and H_2_O_2_. Similarly, edaravone significantly repressed swelling caused by Ca^2+^ load in the mitochondria isolated from the left ventricular tissue of rats. These results suggest that the neuroprotective effect of edaravone could be attributed to the control of mitochondrial permeability transition pore (mPTP) opening [69,70]. Primary human corneal epithelial cells (HCEpiCs) in hyperosmotic media were found to have increased ROS and ATP levels and reduced mitochondrial membrane potential. However, edaravone treatment attenuated these mitochondrial oxidative defects by increasing ATP levels and the mitochondrial membrane potential, preventing apoptosis by reducing cleaved caspase 3 levels, and preventing the release of cytochrome c. Moreover, the administration of edaravone upregulated Nrf2 and its target genes, including HO-1, GPx-1, and GCLC [71]. Edaravone treatment in rat primary cultured astrocytes repressed the cytotoxicity induced by CO as well as the mitochondria-dependent apoptosis pathway and notably increased the mitochondrial membrane potential [72]. Dexamethasone (Dex)-induced osteoblastic cells showed increased ROS production, lipid peroxidation, mPTP opening, and mitochondrial membrane potential. Edaravone alleviated these damages induced by Dex [73]. It also preserved mitochondrial morphology and suppressed mitochondria-dependent apoptosis under exposure to H_2_O_2_ [74]. Moreover, it increased mitochondrial membrane potential and mitochondrial oxidative stress and inhibited mPTP opening in human umbilical vein endothelial cells injured with iron overload [66]. Doxorubicin treatment-induced excessive ROS production and increased apoptosis. Meanwhile, edaravone treatment prevented the release of cytochrome c from mitochondria to the cytosol [67]. These results suggest that recovery of the mitochondrial function in edaravone-treated ex vivo models can be attributed to edaravone-mediated mechanisms, such as an increase in mitochondrial membrane potential and inhibition of mPTP opening.

### 4.3. In Vivo Models

Edaravone plays a role in preventing cellular toxicity by restoring the function of mitochondria, not only in vitro but also in vivo. Doxorubicin-treated rats present high lipid peroxidation and low mitochondrial-dependent antioxidant activity. However, edaravone co-treatment significantly increased antioxidant activity and decreased lipid peroxidation [75]. Edaravone treatment inhibited the loss of mitochondrial membrane potential induced by lipopolysaccharide and recovered antioxidant activity and MDA levels in mitochondria [76]. In addition, edaravone inhibited the expression levels of p-JAK2, p-STAT3, and p-STAT1, downregulated the mitochondrial apoptosis pathway, and alleviated the reduction of mitochondrial membrane potential [77]. Intermittent hypoxia-induced rats showed decreased antioxidant enzyme levels and increased apoptotic signaling from mitochondria, leading to impaired cognition and hippocampal function. Edaravone treatment reversed these impairments in intermittent hypoxia-induced rats [78]. Edaravone was also found to restore expression of PINK1/Parkin and mitochondrial dynamics-related proteins in cerebral ischemia-reperfusion in rats with middle cerebral artery occlusion. Moreover, edaravone inhibited mitochondrial apoptosis induced by cerebral ischemia-reperfusion in rats and prevented swollen mitochondrial morphology [79,80]. Exposure to manganese in rats decreased complex I activity and increased lipid peroxidation. However, edaravone treatment significantly restored mitochondrial complex I activity and enhanced motor performance [81]. Edaravone attenuated cisplatin- and neomycin-induced swollen mitochondria in zebrafish. Furthermore, edaravone protected against neomycin- and cisplatin-induced hair cell loss by blocking apoptotic cell death [82,83]. Thus, edaravone has been demonstrated to be a potential treatment strategy that affects mitochondria function in disease and non-disease models, both in vitro and in vivo.

**Table 2:** Studies that used edaravone in in vivo and ex vivo models of mitochondrial diseases.

Derived Tissue	Associated Disease	Experimental Model	Action of Edaravone in Mitochondria									Ref.
			ΔY	ATP Level	Peroxidation	ROS	Morphology	Fission/Fusion	Biogenesis	mPTP Opening	Apoptosis	
Ex vivo												
Brain	AD	Amyloid-β-treated microglia	Increase	-	-	Decrease	-	-	-	-	-	[68]
		CO-induced rat primary cultured astrocytes	Increase	-	-	-	-	-	-	-	Inhibit	[72]
		Ca^2+^- and H_2_O_2_-induced rat brain	-	-	-	Decrease	Preserve	-	-	-	-	[69]
Cornea		Primary human corneal epithelial cells in hyperosmotic media	Increase	Increase	-	-	-	-	-	-	-	[71]
Bone, Muscle		Dexamethasone-induced primary osteoblast	Increase	-	-	-	-	-	-	Inhibit	-	[73]
Embryo		H_2_O_2_ exposed cortical neuron in rat embryos	-	-	-	-	Preserve	-	-	-	Inhibit	[74]
Heart		Ischemia by left coronary artery occlusion and reperfusion in rat	-	Increase	-	-	Preserve	-	-	Inhibit	Inhibit	[70]
In vivo												
	Myocardial infarction	Doxorubicin-treated rat	-	-	-	Up-regulation of antioxidant enzymes	-	-	-	-	-	[75]
	Obstructive sleep apnea-hypopnea syndrome	Intermittent hypoxia-induced rat	-	-	-	Up-regulation of antioxidant enzyme	-	-	-	-	-	[78]
	Ischemic stroke	Cerebral ischemia-reperfusion in middle cerebral artery occlusion rat	-	-	-	-	-	Up-regulation of Drp1 and Opa1	-	-	Inhibit	[79]
		Manganese-induced rat	-	Recovery of the ETC complex I activity	Decrease	-	-	-	-	-	-	[81]
	Acute kidney injury	Lipopolysaccharide-induced rat	Increase	-	Decrease	Up-regulation of antioxidant enzymes	-	-	-	-	-	[76]
		Kidney ischemia-reperfusion injury rat	Increase	-	-	-	-	-	-	-	Inhibit	[77]
		Reperfusion injury rat	-	-	-	-	Preserve	-	-	-	-	[80]
		Cisplatin-induced zebrafish	-	-	-	-	Preserve	-	-	-	-	[82]
		Neomycin-induced zebrafish	-	-	-	-	Preserve	-	-	-	-	[83]

## 5. Conclusions

**Figure 1:**
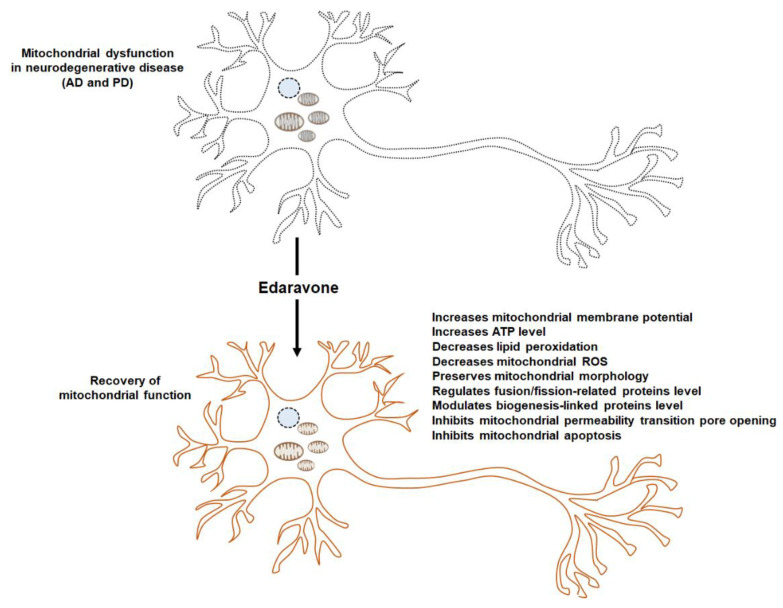
Edaravone modulates mitochondrial function. Neurodegenerative diseases, including AD and PD are characterized by mitochondrial dysfunction. Treatment with edaravone restores mitochondrial functions through various mechanisms, such as the recovery of mitochondrial membrane potential, ATP levels, and mitochondrial morphology, and regulation of mitochondrial fusion/fission and mitochondrial biogenesis-related genes.

Edaravone, a free radical scavenger, ameliorates oxidative damages in various neurodegenerative diseases. Oxidative stress and mitochondrial dysfunction are relevant to neuronal disease, especially ALS. One known mechanism is the anti-oxidant activity of edaravone through the Nrf2 signaling pathway. Furthermore, edaravone exerts a mitochondrial protective effect in neurodegenerative diseases, including AD and PD (Figure 1). In this review, we focused on the mitochondrial protective effect of edaravone, the underlying mechanisms, and the potential targets for its mode of action. Despite considerable evidence of the role of edaravone in mitochondria, the precise mechanism of action remains unclear, and the usage of edaravone in disease therapy is still limited. One of the features of ALS pathogenesis is mitochondrial dysfunction, including impaired mitochondrial morphology, imbalanced mitochondrial dynamics, and reduced ATP levels. While it has been demonstrated that edaravone restores mitochondrial dysfunction in other diseases, the evidence for the same in ALS has not been reported. Clinical research and patient data on edaravone can be leveraged for its therapeutic applications in not only neurodegenerative diseases, including ALS but also other diseases because mitochondrial dysfunction is a characteristic feature of various diseases. More studies are required on the detailed mechanism of edaravone efficiency with respect to mitochondria and to investigate therapeutic applications in various human diseases. In addition, a new initiative in terms of drug repurposing can be taken up for the management of ALS.
